# Lipid droplets contribute myogenic differentiation in C2C12 by promoting the remodeling of the acstin-filament

**DOI:** 10.1038/s41419-021-04273-8

**Published:** 2021-11-23

**Authors:** Yanjie Tan, Yi Jin, Pengxiang Zhao, Jian Wu, Zhuqing Ren

**Affiliations:** 1 Key Laboratory of Agriculture Animal Genetics, Breeding and Reproduction of the Ministry of Education & Key Laboratory of Swine Genetics and Breeding of the Ministry of Agriculture and Rural Affairs, College of Animal Science, Huazhong Agricultural University, 430070 Wuhan, Hubei P. R. China; 2 Institute of Biomedical Sciences, Key Laboratory of Animal Resistance Biology of Shandong Province, College of Life Sciences, Shandong Normal University, 250014 Jinan, Shandong China; 3 Hubei Hongshan Laboratory, Wuhan, Hubei, China

**Keywords:** Cell migration, Organelles

## Abstract

Lipid droplet (LD), a multi-functional organelle, is found in most eukaryotic cells. LDs participate in the regulation of many cellular processes including proliferation, stress, and apoptosis. Previous studies showed the athlete’s paradox that trained athletes accumulate LDs in their skeletal muscle. However, the impact of LDs on skeletal muscle and myogenesis is not clear. We discovered that C2C12 myoblast cells containing more LDs formed more multinucleated muscle fibers. We also discovered that LDs promoted cell migration and fusion by promoting actin-filaments remodeling. Mechanistically, two LD-proteins, Acyl-CoA synthetase long chain family member 3 (ACSL3) and lysophosphatidylcholine acyltransferase 1 (LPCAT1), medicated the recruitment of actinin proteins which contributed to actin-filaments formation on the surface of LDs. During remodeling, the actinin proteins on LDs surface translocated to actin-filaments via ARF1/COPI vesicles. Our study demonstrate LDs contribute to cell differentiation, which lead to new insight into the LD function.

## Introduction

Lipid droplets (LDs) are multi-functional organelles in the cell [1], consisting of a neutral lipid core and a monolayer of phospholipid membranes. Previous studies suggest that LDs are deeply involved in biological processes such as cellular resistance to oxidative stress [2–4], inflammation and immunity [5], and lipid synthesis and metabolism [6, 7]. LDs provide necessary lipid components to cell proliferation including synthesis of membranes and production of metabolic energy [8–10]. LDs perform multiple functions by interacting with other organelles, such as endoplasmic reticulum, mitochondria, peroxisome, and nucleus. For example, LDs interact with the endoplasmic reticulum to regulate cellular lipid synthesis [6]. The contact between LDs and the endoplasmic reticulum and the formation of “lipidic bridges” allows the phospholipid membrane of LDs to link to the outer membrane of the endoplasmic reticulum [11]. Many proteins, such as triglyceride synthases (DGAT2 and GPAT4), are transferred from the endoplasmic reticulum to the LD surface due to the fluidity of the membrane, which accelerates the growth of LDs and allows them to store more lipids [6]. In addition, during LD-mitochondrial interactions, LDs come into contact with mitochondria and fatty acids from LDs are rapidly transferred to the mitochondria for oxidative degradation, providing large amounts of energy [12].

LDs have also been found to interact with the microfilament and microtubules [13]. Recent studies have shown that many LDs are distributed along microtubules [14] and can move unidirectionally or bidirectionally along microtubules [15]. This interaction between LDs and microtubules is important for the LD motility, which allows LDs to interact with other organelles and for the distribution of LD and peroxisomes in the cell [14, 16]. LDs are also found to interact with microfilaments. For example, NMIIa and FMNL1 mediate the assembly of F-actin on the surface of LDs, which in turn affects LD volume and regulates lipid storage in the organism [17]. In addition, proteins of another class of cytoskeletal system (Septin), septin9 [18] and septin11 [19], can regulate LD morphology and growth by regulating the assembly of microfilament microtubules, thereby controlling triglyceride storage and influencing body lipid metabolism [20, 21].

In the present study, we sought to determine the role of LDs and clarify the potential regulatory mechanism of LDs in myoblast differentiation. We show that LDs promote the migration and fusion of myoblast in myogenesis. We provide evidence that LDs regulate actin-cytoskeleton remodeling via buffering the actinin proteins. Our results also suggest the potential link between LDs and muscle development and injury-regeneration.

## Materials and methods

### Cell lines

The C2C12 cell line was purchased from CoWin Biosciences (#CW2915F, Beijing, China). The STR profiling and test of mycoplasma contamination can be provided if required.

### Antibodies

The details are provided in SI Materials and Methods.

### Regents

The details are provided in SI Materials and Methods.

### Strain and plasmid

Trans 5α was purchased from Transgen Biotech (Transgen, Beijing, China). Plasmids including pCMV-C-EGFP (#D2626), pCMV-C-DsRed (#D2624) and pCMV-N-FLAG (#D2722) were purchased from Beyotime (Beyotime, Nanjing, China).

### Cell culture and transfection

The C2C12 cells were cultured in Dulbecco’s Modified Eagle Medium (DMEM; HyClone, Logan, UT, USA) with 10% fetal bovine serum (FBS; #SH30396.03, Hyclone, Canada), 100 unit/mL penicillin, and 100 μg/mL streptomycin in dishes at 37 °C, in a humidified atmosphere with 5% CO_2_. For transfection, a total of 2 μL of Lipofectamine® 2000 transfection reagent (11668-019, Thermo Fisher) diluted in 25 μL of Opti-MEM (51985-034, Thermo Fisher) was prepared. In addition, 1 μg of plasmid was diluted with 25 μL of Opti-MEM and incubated for 5 min at room temperature. The 50 μL mixture of Lipofectamine2000 and plasmids was added into one well (24-well plate). After 5–6 h, the medium was renewed and the cells were incubated for 24–48 h for further use in the following experiments. All analyses were done with three biological replications (three wells of cells per replication).

### C2C12 differentiation

To induce cell differentiation, the C2C12 cells were transferred to DMEM containing 2% horse serum (Gibco) (differentiation medium, DM). All cells were grown to near confluence (90%) before the induction of differentiation.

### Protein mass spectrometry

The protein mass spectrometry detection for this study was commissioned by Novogene Technology Co., Ltd., Beijing, China. In brief, protein alkylation reduction and enzymatic hydrolysis were first performed, followed by peptide desalting, and mass spectrometric detection. The obtained mass spectrometry data were subjected to further quality analysis and protein function annotation for further cluster analysis. For the testing methods and specific parameter settings, we can provide detailed instructions if necessary. The analyses were done with three independent samples replications.

### Lipid droplets isolation

In our previous research, we performed the nuclear separation of cells. In this study, we focused on the function of cytoplasmic lipid droplets. Therefore, we first used the cytoplasmic separation kit to separate the cytoplasmic group, and then we isolated and purified the lipid droplets in the cytoplasm according to Ding et al.’s method [22]. Briefly, we added the cytoplasmic components to buffer A, then ultracentrifuging (185,000×*g*) for 2 h, and taking the upper lipid droplets. The lipid droplet components obtained after ultracentrifugation were washed three times with buffer B, each with a 15,000×*g* centrifugation, and finally the supernatant was discarded. The obtained lipid droplet fractions were used to extract proteins using a protein lysate.

### Western blot

The details are provided in SI Materials and Methods.

### Microfilament marking

In this study, TRITC Phalloidin (#40734ES75, 300T, YEASEN, Shanghai, Chain) was used to label the microfilament cytoskeleton in cells. Briefly, cells were fixed with 4% paraformaldehyde for 30 min. The fixed cells were then incubated with TRITC Phalloidin for 10 min at room temperature. The staining solution was discarded, washed three times with PBS, and the slides were mounted and observed.

### Lipid droplet marking

Lipid droplet marking was performed as reported previously [23]. The cell slides were fixed with 4% paraformaldehyde for 15 min at room temperature. The slides were stained with BODIPY 493/503 (#D3922, Invitrogen, Carlsbad, CA, USA) for 10 min at 37 °C and were then stained with DAPI for 10 min at 37 °C. After washing three times with PBS for 10 min each, the slides were sealed with an anti-fluorescent quenching solution (#P36961, ProLong™ Diamond Antifade Mountant, Invitrogen, Thermo Fisher, USA) for confocal microscopic observation (63× oil lens, BODIPY FL and DAPI channels, Zeiss LSM 800, Germany).

### Cytochalasin treatment

In this study, cytochalasin D was used to disaggregate the microfilaments and destroy the intracellular microfilament cytoskeleton. Briefly, we discarded the original cell culture medium, washed it twice with PBS, added 10 μmol/L Cytochalasin D (#C102396, CAS: 22144-77-0, aladdin, Shanghai, China), and then incubate at 37 °C for 2 h.

### Oleic acid medium treatment

Oleic acid treatment was carried out as described in a previous study [23]. For oleic acid treatment, a 20 mM oleic acid phosphate buffer saline (PBS) mixture and 20% FA-free bovine serum albumin (BSA) medium were prepared, and both media were heated in a 70 °C water bath for 30 min. Finally, the media were mixed. The 10 mM oleic acid-BSA mixture was added to the cell cultural medium at a ratio of 1:49 (v:v). The cells were then either seeded on slides or on plates that had been washed three times using PBS. Then, 1 mL of the oleic acid medium was added to the well and the cells were cultured for 12 h.

### Subcellular components isolation

The protein of subcellular organelle components was isolated with the subcellular Structure Protein Extraction Kit (#C500073, Sangon, Shanghai, China). The details are provided in SI Materials and Methods.

### Plasmid construction

Plasmid construction was performed as reported previously [23]. The details are provided in SI Materials and Methods.

### qPCR assay

Real-time PCR was performed as reported previously [23]. Primer sequences are shown in SI Table S1. The details are provided in SI Materials and Methods.

### Silver staining

Silver staining was performed according to the manual (#P0017S, Beyotime, Nanjing, China). The details are provided in SI Materials and Methods.

### Immunoprecipitation and co-immunoprecipitation

Immunoprecipitation and nuclear co-precipitation were performed according to the manufacturer’s instructions (Protein A/G beads, #B23201, bimake, Shanghai, China). The details are provided in SI Materials and Methods.

### Cellular immunofluorescence

Cellular immunofluorescence analysis was performed as previously described [24]. The following primary antibodies were used: Mouse anti-MYHC (Santa Cruz Biotechnology; 1:200 dilution) and rabbit anti-FLAG (#20543-1-AP, Proteintech, Wuhan, China; 1:100 dilution). The secondary antibodies included anti-rabbit Cy3-IgG (Abclonal, Wuhan, China) and anti-mouse Cy3-IgG (Abclonal, Wuhan, China). All analyses were done with three biological replications (three wells of cells per replication).

### Live cell workstation

Cells were seeded on a 3.5 cm glass-bottom cell culture dish (Ibidi, 80466). Cells were treated with OA or BSA (control) for 12 h. Real-time visualization was made by differential interference contrast (DIC) microscopy, utilizing the Zeiss inverted fluorescence microscope within an incubated chamber (37 °C, 5% CO_2_). Images were captured at 15 min intervals for 2 days.

### Fluorescence images analysis

The ImageJ software was utilized to analyze the co-localization. Briefly, the image was split into the red/green/blue channels. Then, the fluorescence intensity was analyzed, along with the marked line. The values of different channels were plotted in one graph.

### Statistical analysis

All quantitative experiments were evaluated for statistical significance using the software GraphPad Prism v.5.0 (GraphPad Software, Inc. 7825 Fay Avenue, Suite 230 La Jolla, CA 92037 USA). Because the sample size in the experiment was small, Wilcoxon–Mann–Whitney nonparametric tests were employed. The statistical significance (*p*-value) is indicated (*p < 0.05; **p < 0.01). All analyses were done with three biological replications (three samples per replication).

### Statements of approval

We confirm that all methods were performed in accordance with the relevant guidelines and regulations of Ethics Committee of Huazhong Agricultural University.

## Results

### LDs promote myoblast to form multinucleated myotubes

**Fig. 1:**
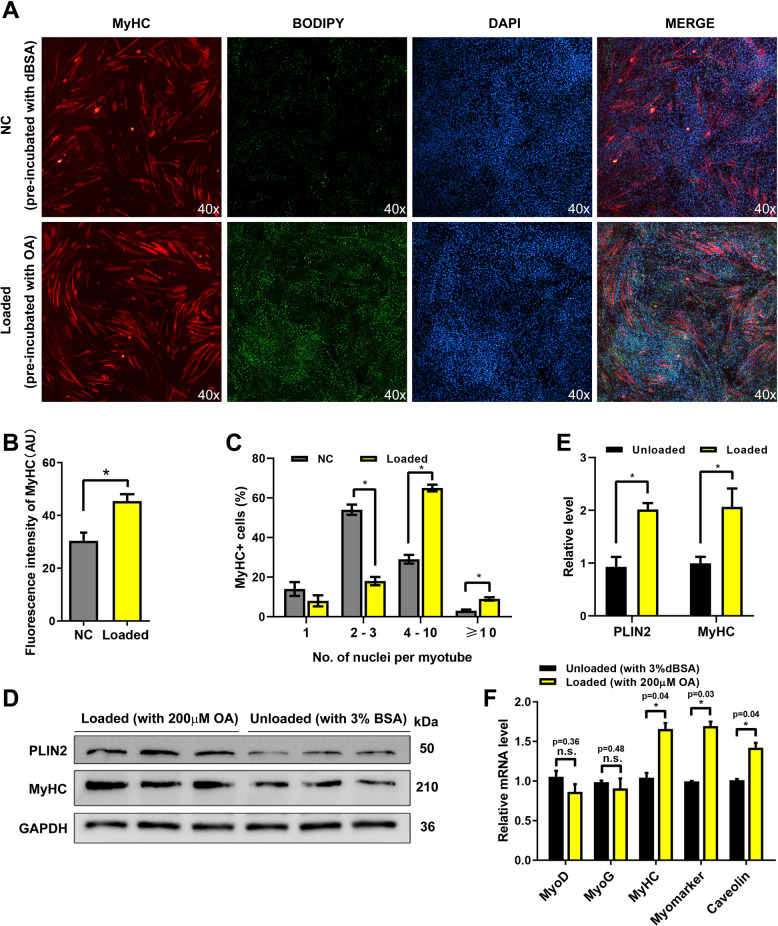
LDs promote myoblast to form multinucleated myotubes. **A** The immunofluorescence detection of MyHC in the loaded (pre-incubated with the OA medium before differentiation) and unloaded cells (pre-incubated with BSA medium). The LDs were stained by BODIPY (green). The nucleus were stained by DAPI. **B** The fluorescence intensity analysis of MyHC. **C** Fusion rate analysis of **A**. **D** PLIN2 and MyHC levels in loaded and unloaded cells after 4 days of differentiation. **E** Gray value analysis of western blotting. **F** The mRNA levels of MyoD, MyoG, MyHC, Myomaker, and Caveolin were detected by qPCR in loaded cells and control cells. *p < 0.05; n.s. not significant. Results are from three technical repeats (*N* = 3) for a representative of three biological repeats (*N* = 3).

To determine whether LDs play a role in myogenesis, we examined the formation of multinucleated myotubes in C2C12 myoblast with more or less LDs. The high-LD-content myoblast (named loaded cells) were constructed by incubation with 200 μM oleic acid for 12 h before differentiation (the control group used 3% BSA). Then the fresh differentiation medium was replaced to induce the myoblast differentiation. Four days later, the shape and number of myotubes were both detected by immunofluorescence. The results showed that the number of myotubes and the fusion rate were significantly higher in the loaded cells (Fig. 1A–C). Additionally, the myosin heavy chain (MyHC) expression was significant upregulate (~1.6-fold) in loaded cells (Fig. 1D, E). Furthermore, the expressions of myogenic differentiation 1 (MyoD) and myogenin (MyoG), the important transcription factors of myogenesis, were not changed (Fig. 1F).

### LDs promote migration and fusion of myoblast

**Fig. 2:**
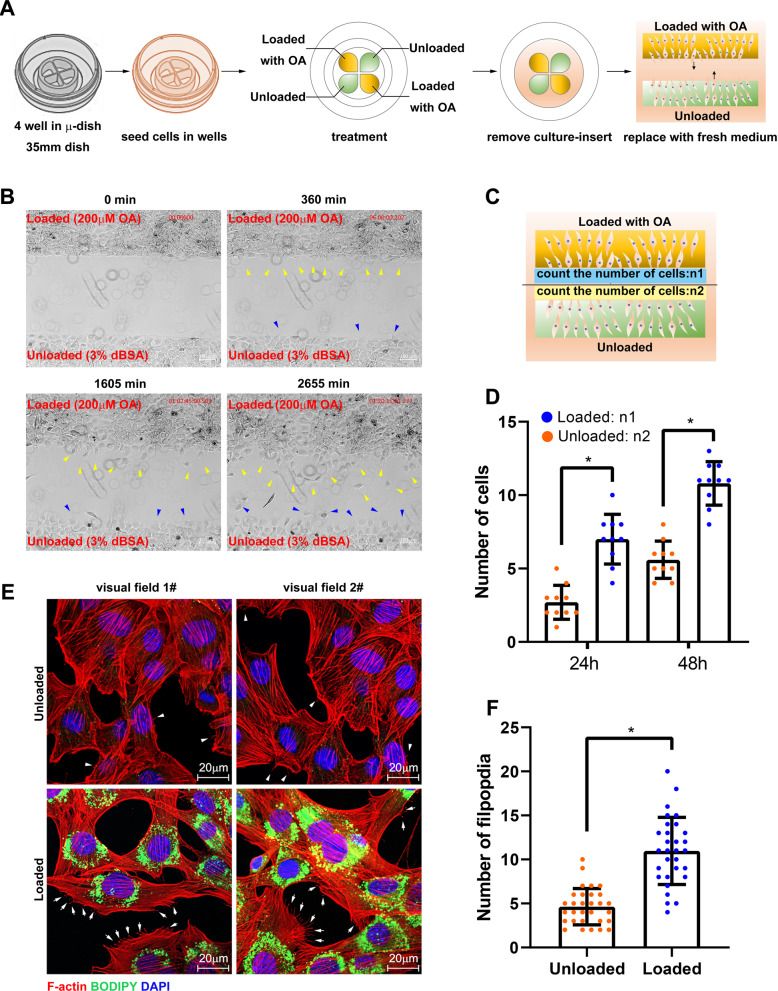
LDs promote migration and fusion of myoblast. **A** The schematic diagram of cell migration assay. C2C12 cells were seeded in the four wells in a 35-nm dish. After treatment, the culture insert was removed, then the dish was observed in a live cell workstation. **B** The images of cell migration at 0, 360, 1605, and 2655 min. The yellow arrows indicate the loaded cells under migration and the blue arrows indicate unloaded cells under migration. **C** Schematic diagram of counting the number of migrating cells. **D** The number of migrating cells at 24 and 48 h. **E** The microfilaments and LDs were marked in loaded and unloaded cells. The arrows indicate the pseudopodia in loaded cells and the arrow head indicate the pseudopodia in unloaded cells. **F** The number of filpopdia in **E** (*N* = 30). *p < 0.05. Results are from three technical repeats (*N* = 3) for a representative of three biological repeats (*N* = 3).

Since LDs cannot affect myogenic regulatory factors, we then examined the migration activity of loaded and control myoblast by scratch assay (Fig. 2A). The results showed the loaded cells formed more filopodia and lamellipodia as compared to unloaded cells at 360 min. Subsequently, more loaded cells were migrated as compared to the unloaded cells at 1605 and 2655 min (Fig. 2B and movie S1). The number of cells with migration was counted, which showed the migration capacity of loaded cells was enhanced (Fig. 2C, D). Furthermore, the F-actin was stained by rhodamine phalloidine, which indicated the loaded cells formed more filopodia and lamellipodia (Fig. 2E, F). Moreover, the expression level of myoblast fusion-associated genes, myomaker, and caveolin were increased significantly in the loaded cells (*p* < 0.05, Fig. 1E).

### LDs accelerate microfilament remodeling

**Fig. 3:**
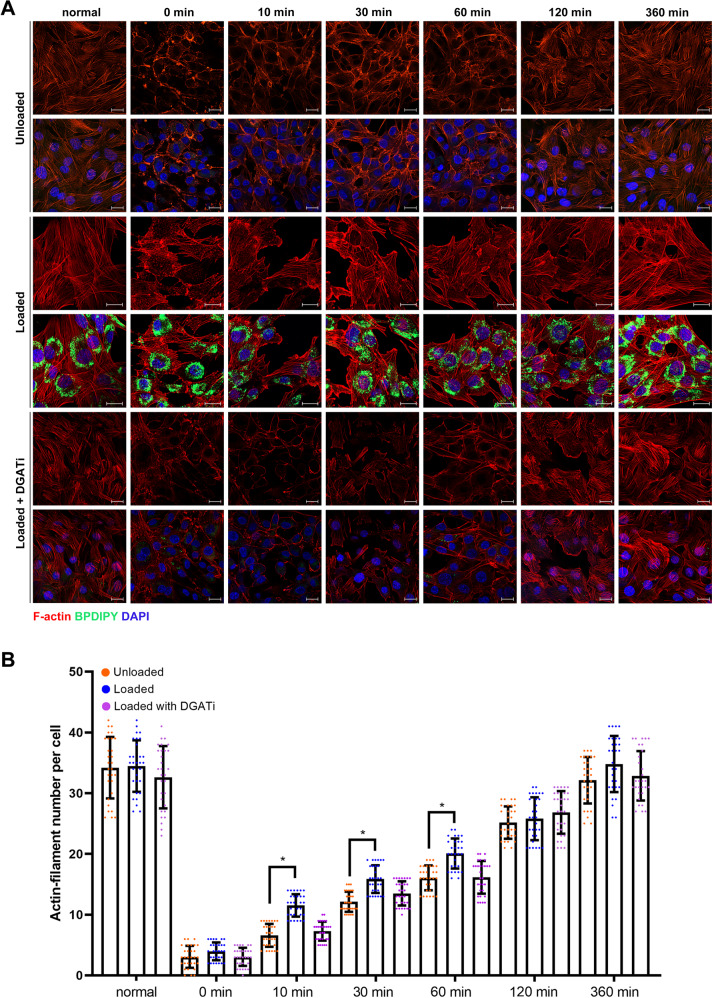
LDs accelerate microfilament remodeling. **A** The rate of actin-filament remodeling. C2C12 cells were pre-treated with 400 μM of oleic acid (OA, named loaded cells) or 3% bovine serum albumin (BSA, control, named unloaded cells). For DGATi treatment, the inhibitor of DGAT1 and DGAT2 was added into medium for 12 h before OA treatment, bar, 20 μm. **B** The count of microfilament number per cell. *p < 0.05. Results are from three technical repeats (*N* = 3) for a representative of three biological repeats (*N* = 3).

Since LDs affected myoblast migration and fusion, we then examined the impact of LDs on remodeling rate. Cytochalasin D, as the specific inhibitor of microfilament polymerization, can inhibit cytoskeleton remodeling. C2C12 cells were treated with cytochalasin for 2 h and microfilaments were then labeled with rhodamine-phalloidin. The microfilament structures were completely destroyed, and the rhodamine signals were diffused (SI Appendix Fig. S1A). Subsequently, fresh medium was changed and the cells were fixed for 0.5, 1, 1.5, 2, 4, and 6 h, respectively. The results showed that microfilaments (peripheral stress fiber) were formed at the edges of cells at 0.5, 1.5, and 2 h (SI Appendix Fig. S1A). The number of new polymerized microfilaments returned to a normal level after 2 h (SI Appendix Fig. S1B). Interestingly, the number of cellular LDs increased during the microfilament polymerization process (SI Appendix Fig. S1A, C). We then examined the remodeling rate in loaded cells and control cells. The results showed that more actin-filaments were formed in loaded cells at 10, 30, and 60 min than in control cells (Fig. 3A, B). In order to confirm the effect of LD on remodeling rate, instead of OA, the C2C12 cells were treated with DGAT1 and DGAT2 inhibitor before OA treatment, which could abrogate the formation of LD. The result showed that the rate of remodeling was not accelerated in cells with DGATi (Fig. 3A, B).

### LD proteomic data show many actinin proteins on LDs

Ping sheng Liu’s lab reported the proteome of C2C12 LD previously [25]. Our lab isolated and purified cytoplasmic LDs of HepG2 cells and then analyzed by mass spectrometry. We found total 132 cytoskeleton-related proteins in LD proteome, including 63 microtubule cytoskeleton and 46 actin cytoskeleton proteins (SI Appendix Fig. S2A). Interestingly, the C2C12 LD proteome also showed many actin cytoskeleton proteins on LD surface [25] (see Appendix file of the reference). Some of them were selected for Western blot verification in both HepG2 and C2C12 cells, including the actinin proteins ACTN1, ACTN2, ACTN3, ACTA1, and tubulin TUBA4A (SI Appendix Fig. S2B).

### LD proteins ACSL3 and LPCAT1 recruit actinin by binding SR protein domains

**Fig. 4:**
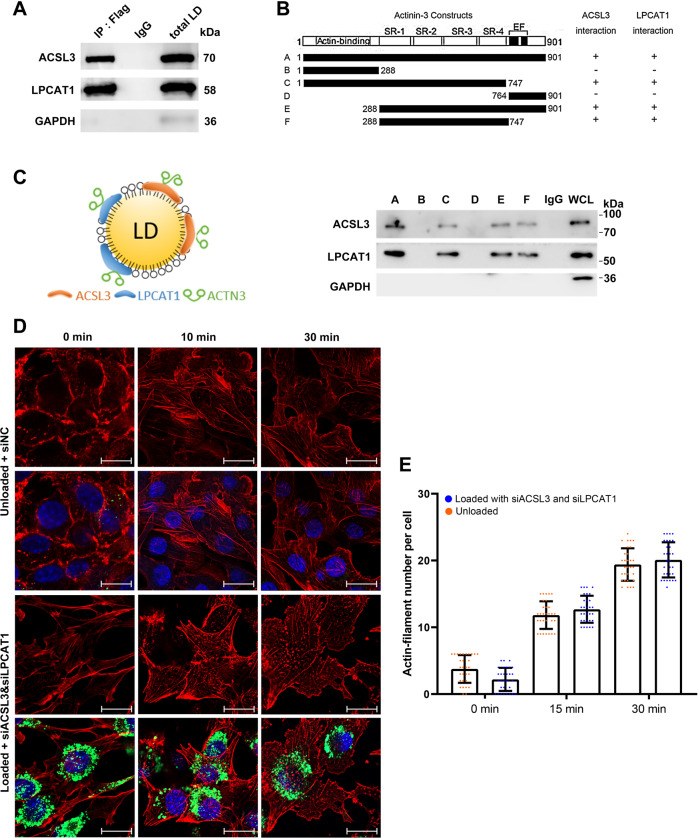
LD proteins ACSL3 and LPCAT1 recruit actinin by binding SR protein domains. **A** Co-IP verification of ACTN3-ACSL3 and ACTN3-LPCAT1 interactions. **B** ACTN3 was truncated according to the domain. The Co-IP detection of the interactions of ACSL3/LPCAT1 and different ACTN3 domains. **C** The schematic diagram of ACTN3 recruited on LD. **D** The rate of actin-filament remodeling in loaded cells with siACSL3 and siLPCAT1 and unloaded cells, bar, 20 μm. **E** The count of microfilament number per cell. *p < 0.05. Results are from three technical repeats (*N* = 3) for a representative of three biological repeats (*N* = 3).

Actinin protein contains three protein domains, namely, the actin-binding domain, spectrin repeat (SR) domain, and EF domain. There are no LD-targeting sequences, such as a giant hydrophobic helix. Therefore, actinin is not a natural LD-targeting protein. To determine how actinin recruits on LDs surface, we performed the immunoprecipitation assay to find the LD-proteins that bind to actinin proteins in C2C12 (SI Appendix Fig. S3A). The protein silver staining image showed large differences in bands between the LD fraction and whole cell lysate (SI Appendix Fig. S3B). The mass spectrometry result showed that ACTN3 binds to many microfilaments, microtubules, and intermediate fibrin (SI Appendix Fig. S3C), and, additionally, the molecular functions and protein domains were also compatible with cytoskeleton-related proteins (SI Appendix Fig. S3D). Furthermore, two LD-proteins, ACSL3 and LPCAT1, were found in the data (SI Appendix Fig. S3C). The two proteins are well-known LD-targeting proteins because they contain LD-targeting structures [26–28]. We then verified that ACTN3 binds to ACSL3 and LPCAT1 via a co-immunoprecipitation assay in C2C12 cells (Fig. 4A). Furthermore, we confirmed the binding effect by using tag vectors in C2C12 cells (SI Appendix Fig. S3E, F). In order to investigate the binding domain of ACTN3, we constructed six different fragments, A to F, containing the FLAG tag according to the domain of ACTN3 (Fig. 4B). The results showed that the A, C, E, and F fragments could bind to ACSL3 and LPCAT1, which indicated the SR domain was the key protein domain for ACTN3 binding to ACSL3 and LPCAT1 (Fig. 4B). In order to further detect whether ACSL3 and LPCAT1 are important factors for ACTN3 localization on LDs, we interfered with the expression of ACSL3 and LPCAT1 by RNAi. Then, the LD-ACTN3 level was detected by Western blotting. The results showed that ACSL3 and LPCAT1 levels were inhibited, and that the LD-ACTN3 level decreased significantly (*p* < 0.05) (SI Appendix Fig. S3G). Therefore, we drew the model to show the binding effect between ACTN3 and ACSL3/LPCAT1 (Fig. 4C). We further investigated whether ACTN3 recruitment on LDs affected their stability through half-life assay. The result indicated ACTN3 degradation was slower in loaded cells than unloaded cells (SI Appendix Fig. S3H). We then detected the affect of ACSL3/LPCAT1 knockdown on the actin-filament remodeling. The result showed that the rate of remodeling did not changed in loaded C2C12 cells with ACSL3/LPCAT1 knockdown (Fig. 4D, E).

### Transfer of actinin from LD surface to microfilament during microfilament remodeling

**Fig. 5:**
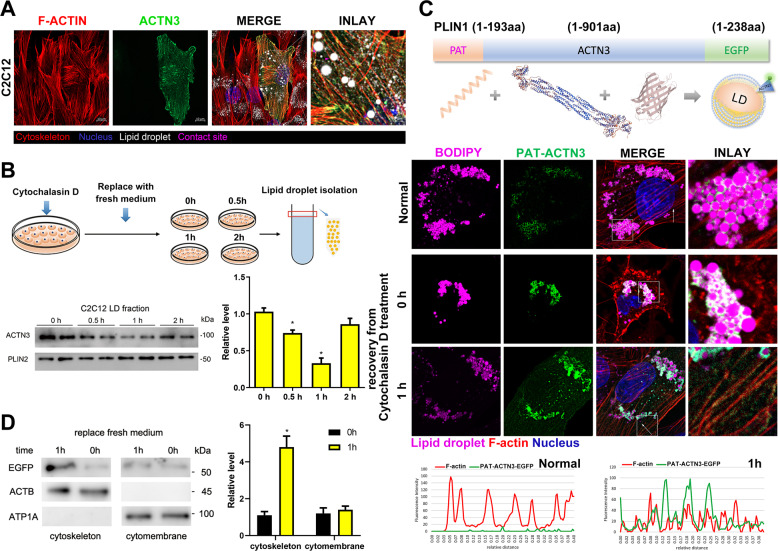
Transfer of actinin from LD surface to microfilament during microfilament remodeling. **A** Co-localization of ACTN3, microfilaments, and LDs in C2C12 cells. **B** The C2C12 cells were treated with cytochalasin D for 2 h. Then, the medium was replaced with a fresh medium. The LDs were isolated at 0 h, 0.5 h, 1 h, and 2 h, respectively. The ACTN3 levels were detected by Western blotting. **C** The LD-targeting ACTN3 plasmid was constructed by inserting a PAT domain (PLIN1 1–193aa) at the N-terminus of ACTN3. The co-localization of PAT-ACTN3-EGFP, LDs, and microfilaments in C2C12 cells was detected by a co-confocal microscope. The fluorescence intensity, along with the arrow line, is plotted in the below graphs. **D** The C2C12 cells (expressing PAT-ACTN3-EGFP) were treated with cytochalasin D for 2 h, then the medium was replaced with a fresh medium. The subcellular components were isolated at 0 and 1 h, respectively. The levels of EGFP were detected. ACTB was utilized as the microfilament reference protein and ATP1A was utilized as cytomembrane reference protein. *p < 0.05. Results are from three technical repeats (*N* = 3) for a representative of three biological repeats (*N* = 3).

Actinin proteins are involved in crosslinking actin-containing thin filaments, are important to the stability and activity of actin-filaments. We further investigated the role of actinin proteins on LDs surface. Firstly, we confirmed the localization of ACTN3 on LDs surface through laser confocal microscope and western blot. The results showed ACTN3 co-localized to whole microfilament (including ventral stress fibers, back stress fibers, peripheral stress fibers, and actin cap stress fibers) in C2C12 cells (Fig. 5A). We then detected the level of LD-ACTN3 during actin-cytoskeleton remodeling. After treating the cells with cytochalasin D for 2 h, the cells were washed by phosphate buffer saline (PBS) twice and then changed into a fresh medium, and the cells were collected for LD isolation at 0, 0.5, 1, and 2 h, respectively. The results show that LD-ACTN3 decreased at 0.5, 1 h and returned at 2 h (Fig. 5B). In order to elucidate why LD-ACTN3 decreased during remodeling, we reformed ACTN3 to be a LD-targeting protein through inserting a PAT protein domain (1–193aa of PLIN1, a giant amphipathic helix) at the N-terminus of ACTN3-EGFP (Fig. 5C). Firstly, the total EGFP signals were localized to the surface of LDs, while no EGFP signal was detected on the microfilaments in C2C12 cells (Fig. 5C). After the C2C12 cells were treated with cytochalasin D for 2 h, the microfilament structure was destroyed, and the localization between the LDs and EGFP protein remained unchanged (Fig. 5C). After replacing the fresh medium for 1 h, new microfilaments were formed, and the EGFP signal was positive on the microfilaments, although EGFP signals were still present surrounding the LDs (Fig. 5C). To further confirm the translocation of LD-ACTN3 to actin-filaments, the proteins of subcellular fractions (such as cytoskeletons, cytomembranes) were isolated, and the levels of EGFP protein in the cell membrane and cytoskeleton components at 0 and 1 h were detected. The results show that the level of EGFP in the cytoskeleton protein at 1 h increased (*p* < 0.05, Fig. 5D) and that the level of EGFP in the cytomembrane protein also increased slightly (Fig. 5D).

### ARF1-dependent vesicles mediate the transfer of actinin from LDs to microfilaments

**Fig. 6:**
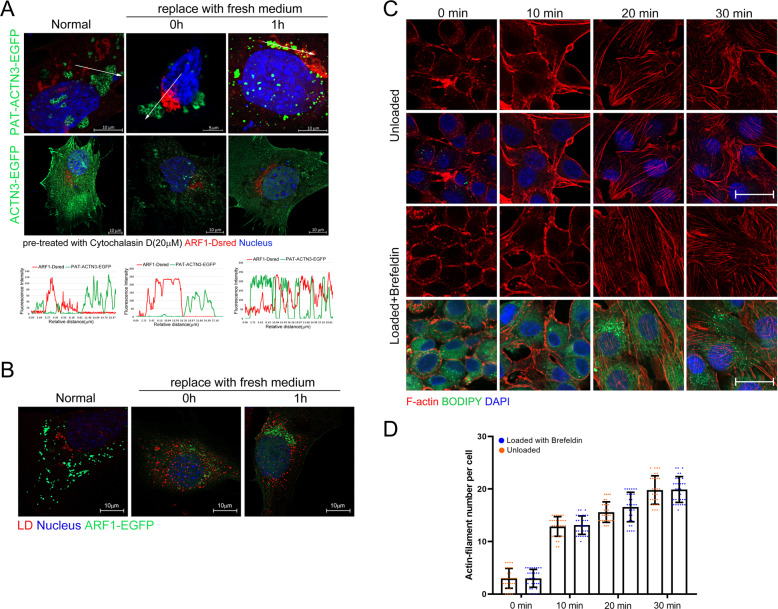
ARF1-dependent vesicles mediate the transfer of actinin from LDs to microfilaments. **A** The C2C12 cells were treated with cytochalasin D for 2 h, then the medium was replaced with a fresh medium. The co-localization of PAT-ACTN3-EGFP and ARF1-DsRed was detected. The fluorescence intensity, along with the arrow line, is plotted in the graph below. **B** The co-localization of LDs and ARF1-EGFP in C2C12 cells was detected. **C** The rate of actin-filament remodeling in loaded cells with brefeldin A treatment and unloaded cells, bar, 20 μm. **D** The count of microfilament number per cell. *p < 0.05. Results are from three technical repeats (*N* = 3) for a representative of three biological repeats (*N* = 3).

In order to investigate how LD-ACTN3 transferred from LD to microfilaments during remodeling, we detected whether the ARF1/COPI membrane vesicle transport system was involved in this process, as previous studies have indicated that the ARF1/COPI system is important to LD morphology and LD-related protein transport [6, 29]. Co-localization analysis of ARF1 vesicles and LD-ACTN3 showed these two factors contact each other during actin-cytoskeleton remodeling but no contact at normal state (Fig. 6A). Furthermore, LDs (marked by LipidTOX) also contacted with ARF1 vesicles (ARF1-EGFP) during actin-cytoskeleton remodeling in C2C12 cells (Fig. 6B). We then used RNAi to interfere with ARF1 expression (SI Appendix Fig. S4A–C), and simultaneously used brefeldin A (to destroy Golgi apparatus) to inhibit the vesicle system. At this time, the ARF1-DsRed signal disappeared (SI Appendix Fig. S4D, E). Subsequently, the result showed LD-ACTN3 cannot translocate to actin-filament during remodeling when ARF1 vesicles were inhibited (SI Appendix Fig. S4F). We then detected whether brefeldin treatment abrogate accelerated microfilament remodeling in OA-loaded cells. The result showed that the rate of actin-filament remodeling in loaded C2C12 cells was not changed after brefeldin treatment (Fig. 6C, D), which indicated the ARF1 vesicles were necessary for LD-ACTN3 translocated.

### LDs modulate microfilament remodeling during C2C12 differentiation

**Fig. 7:**
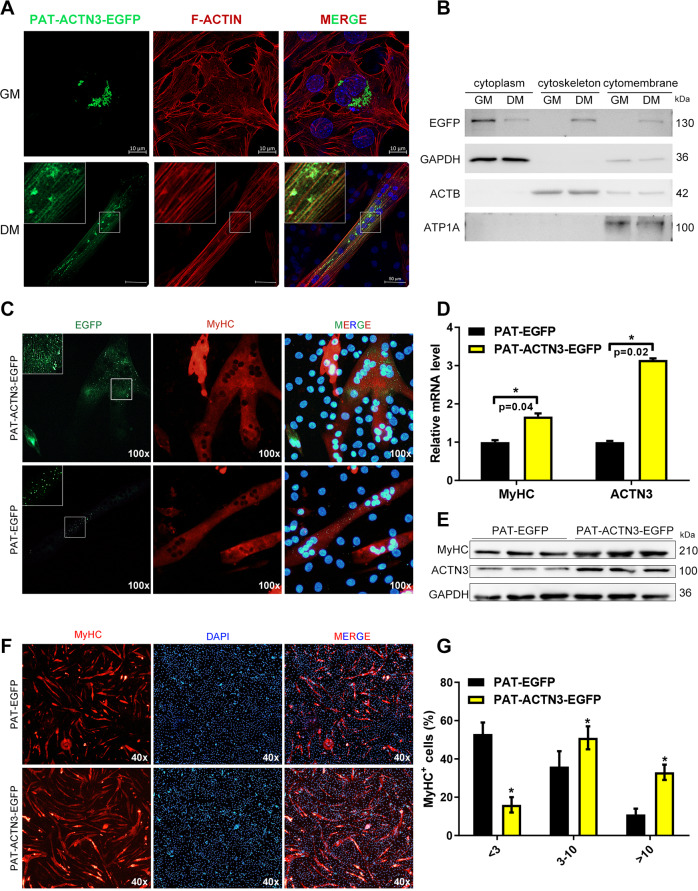
LDs modulate microfilament remodeling during C2C12 differentiation. **A** The co-localization of PAT-ACTN3-EGFP and microfilaments in myoblast and myotubes. **B** The subcellular components were isolated from myoblast and myotubes expressing PAT-ACTN3-EGFP or PAT-EGFP. The protein levels of EGFP were detected by Western blotting. **C** The localization of PAT-ACTN3-EGFP and PAT-EGFP (control) in MyHC+ cells. **D** The mRNA levels of MyHC and ACTN3 were detected by qPCR in cells expressing PAT-ACTN3-EGFP or PAT-EGFP. **E** The protein levels of MyHC and ACTN3 were detected by Western blot in cells expressing PAT-ACTN3-EGFP or PAT-EGFP. **F** The immunofluorescence detection of MyHC in cells expressing PAT-ACTN3-EGFP or PAT-EGFP. **G** The number of nuclei in MyHC+ cells. GAPDH was utilized as the cytoplasm reference protein, ACTB was utilized as the microfilament reference protein, and ATP1A was utilized as the cytomembrane reference protein. *p < 0.05. Results are from three technical repeats (*N* = 3) for a representative of three biological repeats (*N* = 3).

The cytoskeleton is the main source of power in the process of migration and fusion during the differentiation of myoblast. Cell migration and fusion occur during the differentiation of myoblast, and finally multinucleated myotubes are formed (SI Appendix Fig. S5A). In this process, the number of microfilaments in the cell increases and the morphology changes (SI Appendix Fig. S5A). By labeling the microfilament proteins in the cells before and after differentiation, we found that the cell morphology and the number of microfilaments were significantly changed (SI Appendix Fig. S5B, C). A Western blot assay also confirmed that the microfilament-related proteins ACTN1, ACTN2, ACTN3, and ACTA1 increased significantly during C2C12 differentiation (*p* < 0.05, SI Appendix Fig. S5D). We further investigated whether LD-ACTN3 transferred during myoblast differentiation. PAT-ACTN3-EGFP was expressed in myoblast. The co-localization analysis indicated that the EGFP signals were total localized surrounding the LDs (Fig. 7A). Then, the differentiation medium was changed to induce C2C12 differentiation for 4 days. EGFP signals were detected in the microfilaments and LDs of myotubes (Fig. 7A). Moreover, the subcellular components were isolated and the EGFP protein levels in the cytoplasm, cytoskeleton, and cell membrane components of the proliferative and differentiated phases C2C12 were detected. The results showed that the EGFP levels were increased in the cytoskeleton component in differentiated C2C12 cells (Fig. 7B), which suggests that the LD-ACTN3 transferred to cytoskeleton microfilaments during differentiation. For further verification, we marked myotubes by MyHC immunofluorescence. EGFP signals did localize in the microfilaments of myotubes, whereas the control group (expressed PAT-EGFP) showed no signals in microfilaments (Fig. 7C). We further investigated whether PAT-ACTN3-EGFP affected C2C12 differentiation by overexpression. The qPCR and WB results show that MyHC was upregulated in cells with PAT-ACTN3-EGFP overexpression as compared to the control group cells (Fig. 7D, E). Moreover, we detected the number of multinucleated myotubes through MyHC immunofluorescence. The results show that more myotubes were formed in cells with PAT-ACTN3-EGFP overexpression, and, additionally, the number of nuclei in one myotube was higher in cells with PAT-ACTN3-EGFP overexpression also (Fig. 7F, G). In summary, during the differentiation of C2C12 cells, LD-ACTN3 can be transferred to microfilaments and promote cell fusion into multinucleated myotubes.

## Discussion

LDs play an important role in the regulation of cellular lipid metabolism and stress resistance [30]. The LDs themselves are generally not directly involved in the regulation of these biological processes, which they regulate through contact and interaction with other organelles [31]. LDs were reported to interact with microfilament and microtubules. It is well known that LDs are attached to microfilaments and microtubules in cells and can move along the cytoskeleton [13]. Some of the kinetic proteins provide energy for LD movement, making LDs a highly dynamic organelle, which is the basis for interaction with other organelles. Previous studies have shown that microfilament microtubule-related proteins, including NMIII/MYH-9, FMNL1 [17], SEPT9 [18], SEPT11 [19], and SPASTIN [32], can regulate the morphology of LDs. For example, FMNL1 mediates the assembly of NMIIIa and actin microfilaments on LDs, while NMIIIa and microfilaments surround LDs and form a transient foci between the two separated lipid droplets, hindering the fusion and expansion of LDs, thereby reducing the accumulation of triglycerides and enhancing the contact between lipase and LDs, which can promote the breakdown of LDs [17]. Septins are 13-member GTP-binding protein families that are highly conserved from yeast to humans [33], which are closely associated to the cytoskeleton, and also considered to be the fourth cytoskeleton structure [34]. Among them, septin-9 can bind to Ptdlns5P on the surface of LDs and simultaneously bind to microtubules, which can promote the contact between LDs and microtubes and the growth and aggregation of LDs [18]. Our study has validated the existence of multiple microfilament and microtubule-associated proteins on LDs. In addition, the actinins on LDs surface can transfer to microfilament during microfilament remodeling.

We utilized ACTN3 as the research object here to analyze the mechanism of LDs regulating cytoskeletons. ACTN3, as a member of the ACTN family (ACTN1, ACTN2, ACTN3, and ACTN4), can fix microfilaments and maintain the stability of microfilament structure [35–38]. ACTN3 is structurally conserved when compared with other proteins in the ACTNs family. It contains the ACTIN binding domain, four SR (spectrin repeat) domains, and the EF binding domain [39]. Previous studies have reported that natural LD-targeting proteins contain lipid affinity sequences, such as a giant hydrophobic helix structure [28], but ACTNs do not contain such hydrophobic helix structures. Therefore, ACTN3 does not directly bind to lipid droplets through its own protein domain. Therefore, we have revealed that two LD-related proteins, ACSL3 and LPCAT1, are bound to ACTN3 by co-immunoprecipitation. LPCAT1 and ACSL3 have a hydrophobic spiral structure that is compatible with the neutral lipid core of LDs [26, 27] and recruited on the surface of LDs. Through further domain interaction assays, the SR protein domain of ACTN3 was found to interact with ACSL3 and LPCAT1. Therefore, ACTN3 can be indirectly recruited on LDs through its SR domain binding to ACSL3 and LPCAT1. This binding method is also consistent with previous studies, that is, the SR protein domain of ACTNs is usually the platform where it interacts with other proteins [40–43]. We found that PAT-ACTN3 was completely localized on LDs in normal cells and even in depolymerized microfilament cells, and no fluorescent signal was detected on the microfilaments. However, during the microfilament re-polymerization process, fluorescent signals were found on the re-polymerized microfilaments, which suggests that LD-ACTN3 is involved in the microfilament remodeling process. In addition, the phenomenon was also observed in the C2C12 differentiation process.

We considered that the transfer of ACTN3 from LDs to the microfilament is a kind of protein transport process. We found that the ARF1-COPI membrane vesicle transport system played an important role in this transfer process. After interfering with ARF1 expression in cells and using brefeldin A (i.e., dissolving the Golgi apparatus to prevent COPI vesicles) from inhibiting membrane vesicle formation, this transfer process of LD-ACTN3 also disappeared. Therefore, we believe that ARF1-COPI has mediated the transfer of ACTN3 from LDs to the microfilaments during microfilament remodeling. Previous studies have shown that the ARF1-COPI membrane vesicle transport system is very important for LD surface tension and activity. COPI is generated by the Golgi apparatus. In the Golgi apparatus, ARF1 recruits coatomers to the double layer in a GTP-dependent manner [44–46]. In vitro tests have shown that ARF1 and COPI can directly bind to the parent monolayer of phospholipid membranes of artificial LDs. This interaction causes the parent LDs to bud out to form 60-nm nano-LDs [29]. This budding process increases the surface tension of LDs, which makes the parent LDs more likely to react with the surrounding environment (e.g., soluble enzymes or membranes). Other studies have shown that the ARF1-COPI system is an important factor in regulating LD’s contact with the endoplasmic reticulum, and is very important for the transfer of lipid synthesis-related enzymes to LDs [6]. Although our study does not reveal how ARF1-COPI regulates the transfer of ACTN3 from LDs to microfilament, we speculate that this process is related to the ARF1-COPI-mediated nano-LD release process. Moreover, ACTN3 is indirectly recruited on LDs, and it is likely that the phospholipid cluster released by budding leaves the LD surface and then participates in the polymerization of the microfilament. There is no doubt that this process still needs further research and experimental data for support.

A number of studies have reported that LDs are in contact with mitochondria. Skeletal muscle is an oxidatively active tissue with a large number of mitochondria distributed, and electron microscopy results show that LDs are in close contact with mitochondria [47, 48]. Several molecules have been identified that potentially regulate LD-mitochondrial contact, including PLIN5 [49], SNAP23 [50], Mfn2 [51], MIGA2 [52], and VPS13D [53], and deletion of each of these proteins leads to a reduction in LD-mitochondrion contact sites. These molecules may serve to tether LDs to mitochondria, allowing stable connections to form between LDs and mitochondria. A study reported that dynamic mitochondrial-cytoskeletal interactions can facilitate network function and remodeling [54]. The transfer of actinin from LDs to microfilaments may also be a mitochondrial-dependent mechanism. It has been suggested that LDs can follow the movement of other organelles, including mitochondria [15]. The possibility that organelles in contact with each other may be jointly involved in a biological process deserves further investigation.

**Fig. 8:**
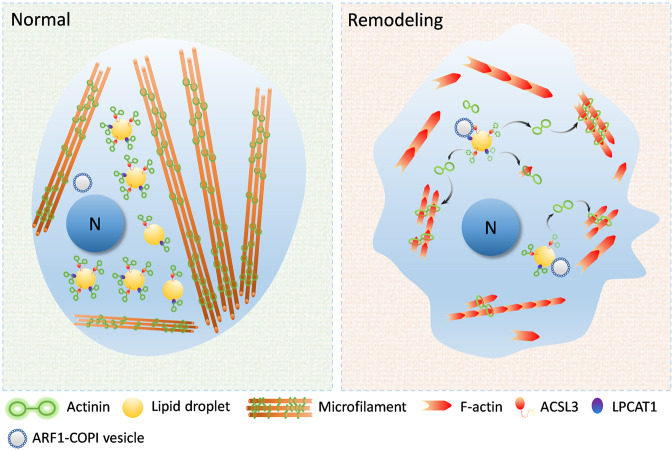
The schematic diagram of LDs modulating microfilament remodeling. Cellular LDs in myoblast can recruit actinins through the LD-related proteins ACSL3 and LPCAT1. When the cellular microfilaments remodeling (e.g., migration, fusion) which promoted contact between LDs and ARF1-COPI vesicles. Meanwhile, the recruited actinins are released from maternal LDs. Thereafter, the released actinins from the LDs were involved the polymerization and remodeling of microfilaments. LDs contribute the process of cellular microfilaments remodeling.

In summary, we have assumed that the model of LDs regulates microfilament remodeling (Fig. 8). Cellular LDs can recruit actinins through the LD-related proteins ACSL3 and LPCAT1. When the cellular microfilaments are “broken down”, the shape of the cells shrink, promoting contact between the LDs and ARF1-COPI vesicles. The contact between the LDs and ARF1-COPI vesicle induces the production of 60 nm nano-LDs. Meanwhile, the recruited actinins were released from maternal LDs, along with these nano-LDs. Thereafter, the released actinins from LDs are involved in the polymerization and remodeling of microfilaments. This function of LD could lead to enhanced migration capacity of cells and promote the process of myogenic cell differentiation. In this study, we identified the effect of LD buffering actinin on myogenic cell differentiation, which provides new ideas for the role of LDs in muscle development as well as in injury repair process.

## Supplementary information

SI Appendix

Supplymentary video S1

## Data Availability

The data used to support the findings of this study are available from the corresponding author upon request.

## References

[1] Walther TC, Farese RV (2012). Lipid droplets and cellular lipid metabolism. Annu Rev Biochem.

[2] Bailey AP, Koster G, Guillermier C, Hirst EMA, MacRae JI, Lechene CP (2015). Antioxidant role for lipid droplets in a stem cell niche of Drosophila. Cell.

[3] Welte MA (2015). How brain fat conquers stress. Cell.

[4] Liu L, Zhang K, Sandoval H, Yamamoto S, Jaiswal M, Sanz E (2015). Glial lipid droplets and ROS induced by mitochondrial defects promote neurodegeneration. Cell.

[5] Karagiannis F, Masouleh SK, Wunderling K, Surendar J, Schmitt V, Kazakov A, et al. Lipid-droplet formation drives pathogenic group 2 innate lymphoid cells in airway inflammation. Immunity. 2020;52:885. 10.1016/j.immuni.2020.04.021 32433951

[6] Wilfling F, Thiam AR, Olarte MJ, Wang J, Beck R, Gould TJ, et al. Arf1/COPI machinery acts directly on lipid droplets and enables their connection to the ER for protein targeting. Elife. 2014;3:e01607. 10.7554/eLife.01607 PMC3913038 24497546

[7] Wilfling F, Haas JT, Walther TC, Farese RV (2014). Lipid droplet biogenesis. Curr Opin Cell Biol.

[8] Martinez-Lopez N, Singh R (2015). Autophagy and lipid droplets in the liver. Annu Rev Nutr.

[9] Schulze RJ, Sathyanarayan A, Mashek DG (2017). Breaking fat: the regulation and mechanisms of lipophagy. Biochim Biophys Acta.

[10] Zechner R, Madeo F, Kratky D (2017). Cytosolic lipolysis and lipophagy: two sides of the same coin. Nat Rev Mol Cell Biol.

[11] Olzmann JA, Carvalho P (2019). Dynamics and functions of lipid droplets. Nat Rev Mol Cell Biol.

[12] Rambold AS, Cohen S, Lippincott-Schwartz J (2015). Fatty acid trafficking in starved cells: regulation by lipid droplet lipolysis, autophagy, and mitochondrial fusion dynamics. Dev Cell.

[13] Welte MA (2009). Fat on the move: intracellular motion of lipid droplets. Biochem Soc Trans.

[14] Herms A, Bosch M, Reddy BJN, Schieber NL, Fajardo A, Ruperez C, et al. AMPK activation promotes lipid droplet dispersion on detyrosinated microtubules to increase mitochondrial fatty acid oxidation. Nat Commun. 2015;6:1–14. 10.1038/ncomms8176 PMC4446796 26013497

[15] Kilwein MD, Welte MA. Lipid droplet motility and organelle contacts. Thousand Oaks:Contact;2019. 10.1177/2515256419895688 PMC6943980 31909374

[16] Lin CP, Schuster M, Guimaraes SC, Ashwin P, Schrader M, Metz J, et al. Active diffusion and microtubule-based transport oppose myosin forces to position organelles in cells. Nat Commun. 2016;7:1–14. 10.1038/ncomms11814 PMC4895713 27251117

[17] Pfisterer SG, Gateva G, Horvath P, Pirhonen J, Salo VT, Karhinen L, et al. Role for formin-like 1-dependent acto-myosin assembly in lipid droplet dynamics and lipid storage. Nat Commun. 2017;8:1–14. 10.1038/ncomms14858 PMC5380971 28361956

[18] Akil A, Peng J, Omrane M, Gondeau C, Desterke C, Marin M, et al. Septin 9 induces lipid droplets growth by a phosphatidylinositol-5-phosphate and microtubule-dependent mechanism hijacked by HCV. Nat Commun. 2016;7:1–19. 10.1038/ncomms12203 PMC4947189 27417143

[19] Moreno-Castellanos N, Rodriguez A, Rabanal-Ruiz Y, Fernandez-Vega A, Lopez-Miranda J, Vazquez-Martinez R (2017). The cytoskeletal protein septin 11 is associated with human obesity and is involved in adipocyte lipid storage and metabolism. Diabetologia.

[20] Bostrom P, Rutberg M, Ericsson J, Holmdahl P, Andersson L, Frohman MA (2005). Cytosolic lipid droplets increase in size by microtubule-dependent complex formation. Arterioscl Throm Vas.

[21] Welte MA, Cermelli S, Griner J, Viera A, Guo Y, Kim DH (2005). Regulation of lipid-droplet transport by the perilipin homolog LSD2. Curr Biol.

[22] Ding Y, Zhang S, Yang L, Na H, Zhang P, Zhang H (2013). Isolating lipid droplets from multiple species. Nat Protoc.

[23] Tan Y, Jin Y, Wang Q, Huang J, Wu X, Ren Z. Perilipin 5 protects against cellular oxidative stress by enhancing mitochondrial function in HepG2 cells. Cells. 2019;8:1241. 10.3390/cells8101241 PMC6830103 31614673

[24] Zhang Y, Li W, Zhu M, Li Y, Xu Z, Zuo B (2016). FHL3 differentially regulates the expression of MyHC isoforms through interactions with MyoD and pCREB. Cell Signal.

[25] Zhang HN, Wang Y, Li J, Yu JH, Pu J, Li LH (2011). Proteome of skeletal muscle lipid droplet reveals association with mitochondria and apolipoprotein A-I. J Proteome Res.

[26] Kassan A, Herms A, Fernandez-Vidal A, Bosch M, Schieber NL, Reddy BJN (2013). Acyl-CoA synthetase 3 promotes lipid droplet biogenesis in ER microdomains. J Cell Biol.

[27] Moessinger C, Kuerschner L, Spandl J, Shevchenko A, Thiele C (2011). Human lysophosphatidylcholine acyltransferases 1 and 2 are located in lipid droplets where they catalyze the formation of phosphatidylcholine. J Biol Chem.

[28] Copic A, Antoine-Bally S, Gimenez-Andres M, Garay CL, Antonny B, Manni MM, et al. A giant amphipathic helix from a perilipin that is adapted for coating lipid droplets. Nat Commun. 2018;9:1–16. 10.1038/s41467-018-03717-8 PMC5889406 29626194

[29] Thiam AR, Antonny B, Wang J, Delacotte J, Wilfling F, Walther TC (2013). COPI buds 60-nm lipid droplets from reconstituted water-phospholipid-triacylglyceride interfaces, suggesting a tension clamp function. Proc Natl Acad Sci USA.

[30] Welte MA, Gould AP (2017). Lipid droplet functions beyond energy storage. Biochim Biophys Acta.

[31] Schuldiner M, Bohnert M (2017). A different kind of love-lipid droplet contact sites. Biochim Biophys Acta.

[32] Papadopoulos C, Orso G, Mancuso G, Herholz M, Gumeni S, Tadepalle N, et al. Spastin binds to lipid droplets and affects lipid metabolism. PLoS Genet. 2015;11:e1005149. 10.1371/journal.pgen.1005149 PMC4395272 25875445

[33] Nishihama R, Onishi M, Pringle JR (2011). New insights into the phylogenetic distribution and evolutionary origins of the septins. Biol Chem.

[34] Mostowy S, Cossart P (2012). Septins: the fourth component of the cytoskeleton. Nat Rev Mol Cell Biol.

[35] Edlund M, Lotano MA, Otey CA (2001). Dynamics of alpha-actinin in focal adhesions and stress fibers visualized with alpha-actinin-green fluorescent protein. Cell Motil Cytoskel.

[36] Otey CA, Carpen O (2004). alpha-actinin revisited: a fresh look at an old player. Cell Motil Cytoskel.

[37] Mills MA, Yang N, Weinberger RP, Vander Woude DL, Beggs AH, Easteal S (2001). Differential expression of the actin-binding proteins, alpha-actinin-2 and-3, in different species: implications for the evolution of functional redundancy. Hum Mol Genet.

[38] Araki N, Hatae T, Yamada T, Hirohashi S (2000). Actinin-4 is preferentially involved in circular ruffling and macropinocytosis in mouse macrophages: analysis by fluorescence ratio imaging. J Cell Sci.

[39] Sjoblom B, Salmazo A, Djinovic-Carugo K (2008). alpha-actinin structure and regulation. Cell Mol Life Sci.

[40] Atkinson RA, Joseph C, Kelly G, Muskett FW, Frenkiel TA, Nietlispach D (2001). Ca2+-independent binding of an EF-hand domain to a novel motif in the alpha-actinin-titin complex. Nat Struct Biol.

[41] Franzot G, Sjoblom B, Gautel M, Carugo KD (2005). The crystal structure of the actin binding domain from alpha-actinin in its closed conformation: structural insight into phospholipid regulation of alpha-actinin. J Mol Biol.

[42] Djinovic-Carugo K, Gautel M, Ylanne J, Young P (2002). The spectrin repeat: a structural platform for cytoskeletal protein assemblies. Febs Lett.

[43] Kusunoki H, Macdonald RI, Mondragon A (2004). Structural insights into the stability and flexibility of unusual erythroid spectrin repeats. Structure.

[44] Faini M, Beck R, Wieland FT, Briggs JAG (2013). Vesicle coats: structure, function, and general principles of assembly. Trends Cell Biol.

[45] Serafini T, Orci L, Amherdt M, Brunner M, Kahn RA, Rothman JE (1991). Adp-ribosylation factor is a subunit of the coat of golgi-derived Cop-coated vesicles—a novel role for a Gtp-binding. Protein Cell.

[46] Donaldson JG, Cassel D, Kahn RA, Klausner RD (1992). Adp-ribosylation factor, a small Gtp-binding protein, is required for binding of the coatomer protein Beta-Cop to golgi membranes. Proc Natl Acad Sci USA.

[47] Bosma M, Sparks LM. Hooiveld GJEJ, Jorgensen JA, Houten SM, Schrauwen P, et al. Overexpression of PLIN5 in skeletal muscle promotes oxidative gene expression and intramyocellular lipid content without compromising insulin sensitivity. Biochim Biophys Acta. 2013;1831:844–52. 10.1016/j.bbalip.2013.01.007 23353597

[48] Shaw CS, Jones DA, Wagenmakers AJM (2008). Network distribution of mitochondria and lipid droplets in human muscle fibres. Histochem Cell Biol.

[49] Olzmann JA, Carvalho P. Dynamics and functions of lipid droplets. Nat Rev Mol Cell Biol. 2018:20;137–155. 10.1038/s41580-018-0085-z PMC6746329 30523332

[50] Jagerstrom S, Polesie S, Wickstrom Y, Johansson BR, Schroder HD, Hojlund K (2009). Lipid droplets interact with mitochondria using SNAP23. Cell Biol Int.

[51] Boutant M, Kulkarni SS, Joffraud M, Ratajczak J, Valera-Alberni M, Combe R (2017). Mfn2 is critical for brown adipose tissue thermogenic function. Embo J.

[52] Freyre CAC, Rauher PC, Ejsing CS, Klemm RW (2019). MIGA2 links mitochondria, the ER, and lipid droplets and promotes de novo lipogenesis in adipocytes. Mol Cell.

[53] Wang J, Fang N, Xiong J, Du Y, Cao Y, Ji WK (2021). An ESCRT-dependent step in fatty acid transfer from lipid droplets to mitochondria through VPS13D-TSG101 interactions. Nat Commun.

[54] Moore AS, Holzbaur ELF. Mitochondrial-cytoskeletal interactions: dynamic associations that facilitate network function and remodeling. Curr Opin Physiol. 2018;3:94–100. 10.1016/j.cophys.2018.03.003 PMC6289269 30555978

