# Supplementary material for: Lipid droplets contribute myogenic differentiation in C2C12 by promoting the remodeling of the acstin-filament
Source: Cell Death Dis. 2021 Nov 23;12(12):1102. doi: 10.1038/s41419-021-04273-8 (PMC8611090; doi:10.1038/s41419-021-04273-8)
Supplement: Supplementary file 1 — SI Appendix [file 41419_2021_4273_MOESM1_ESM.docx]

**Supplementary Information for**

### Lipid droplets contribute myogenic differentiation in C2C12 by promoting the remodeling of the actin-filament

**This PDF file includes:**

Supplementary text

Figures S1 to S5

Tables S1

Legends for Movies S1

SI References

**Other supplementary materials for this manuscript include the following:**

Movies S1

Materials and Methods

**Antibodies.** Following rabbit polyclonal antibodies were used. Rabbit polyclonal anti-PLIN2 (#15294-1-AP), Rabbit polyclonal anti-EGFP (#50430-2-AP), Rabbit polyclonal anti-ARF1 (#10790-1-AP), Rabbit polyclonal anti-ACSL3 (#20710-1-AP), Rabbit polyclonal anti-LPCAT1 (#16112-1-AP), Rabbit polyclonal anti-FLAG (#20543-1-AP) were purchased from Proteintech (Proteintech, Wuhan, China). Rabbit polyclonal anti-ACTN1 (#D121591), Rabbit polyclonal anti-ACTN2 (#D262156), Rabbit polyclonal anti-ACTN3 (#D121486), Rabbit polyclonal anti-TUBA4A (#D110022), Rabbit polyclonal anti-ACTA1 (#D221592), Rabbit polyclonal anti-ACSL3 (#D161226) were purchased from Sangon (Sangon, Shanghai, China). Rabbit polyclonal anti-GAPDH (#AC027) was purchased from Abclonal (Abclonal, Wuhan, China). Following Mouse monoclonal antibodies were used. Mouse monoclonal anti-FLAG (#D190828) was purchased from Sangon (Sangon, Shanghai, China). Mouse monoclonal anti-MYHC (#sc-376157) was purchased from Santa Cruz Biotechnology. Following secondary antibodies were used. Goat anti-Mouse IgG (H+L), Cy3 (#AS008), Goat anti-Rabbit IgG (H+L), Cy3 (#AS007), HRP-labeled Goat Anti-Rabbit IgG (#AS014), HRP-labeled Goat Anti-Mouse IgG (#AS003) were purchased from Abclonal (Abclonal, Wuhan, China).

**Regents.** BODIPY 493/503 was purchased from Invitrogen (#D3922, Invitrogen, Carlsbad, CA, USA). DAPI (#C1002) was purchased from Beyotime (Beyotime, Nanjing, China). TRITC Phalloidin (#40734ES75) was purchased from YEASEN (YEASEN, Shanghai, Chain). Cytochalasin D (#C102396) was purchased from Aladdin (Aladdin, Shanghai, China). M5 ECL Western Blot Kit (#MF-078-01) was purchased from Mei5bio (Mei5bio, Beijing, China). Subcellular Structure Protein Extraction Kit (#C500073) was purchased from Sangon (Sangon, Shanghai, China). ClonExpress II One Step Cloning Kit (#C112-01) was purchased from Vazyme (Vazyme, Nanjing, China). DGAT1 inhibitor (A 922500, #HY-10038) and DGAT2 inhibitor (PF-06424439, #HY-108341) were purchased from MCE (MCE, Shanghai, Chain).

**Western blot.** Western blotting was performed as reported previously^1,2^. Briefly, cells were collected and homogenized in a lysis buffer (#P0013, Beyotime Biotechnology, Nanjing, China). Then, the homogenates were incubated with an SDS-PAGE sample loading buffer (#P0015A, Beyotime Biotechnology, Nanjing, China) at 98 °C for 10 min. Subsequently, the samples were separated by 10% sodium dodecyl sulfate-polyacrylamide gel electrophoresis (SDS-PAGE) and were transferred to a polyvinylidene fluoride (PVDF) membrane (Biorad, USA) using a semidry electrophoretic apparatus. The blocked membranes (#P0252-100mL, QuickBlock™ Blocking Buffer for Western Blot, Beyotime Biotechnology, Nanjing, China) were incubated with antibodies overnight at 4 °C. The blots were extensively washed three times with tris-buffered saline with a Tween 20 (TBST) buffer for 10 min and were then incubated under gentle agitation with the primary antibodies for immunodetection at 37 °C for 1.5 h (diluted in QuickBlock™ Primary Antibody Dilution Buffer for Western Blot, #P0256, Beyotime Biotechnology, Nanjing, China). Then, the blots were extensively washed three times with TBST. Subsequently, the blots were incubated under gentle agitation with the secondary antibodies for immunodetection at 37 °C for 1 h (diluted in QuickBlock™ Secondary Antibody Dilution Buffer for Western Blot, #P0258, Beyotime Biotechnology, Nanjing, China). For detection, the M5 ECL Western Blot Kit (#MF-078-01, Mei5bio, Beijing, China) and a chemiluminescence imaging system (LAS4000, ImageQuant, Germany) were used. All analyses were done with three biological replications (three cell samples per replication).

**Subcellular components isolation.** The protein of subcellular organelle components was isolated with the subcellular Structure Protein Extraction Kit (#C500073, Sangon, Shanghai, China). Briefly, for adherent cells, we washed the cells directly with ice-cold wash buffer twice and aspirated the supernatant. We then resuspended the cells, transferred them to a 2 mL sterile clean centrifuge tube, and washed them again. Then, we extracted the cytosolic proteins. For adherent cells, we added 1 mL of the ice-cold extraction buffer 1 and 10 μL of the protease inhibitor to each T25 culture flask (~1–5 × 10^6^ cells). We then placed the ice bath cells on a decolorization shaker at a medium speed for 5-10 min until 95-100% of the cells were lysed, then centrifuged at 800 g (3000 rpm) at 4 °C for 5-10 min. We carefully aspirated the supernatant and aliquot and stored them at -70 °C. This is the cytosolic protein and soluble cytoskeleton protein extract. The cell membrane and organelle proteins were then extracted. For adherent cells, we added 1 mL of ice-cold extraction buffer 2 and 10 μL of the protease inhibitor to each T25 flask (approximately 5 × 10^6^ cells). We placed the ice bath cells on a destaining shaker at medium speed for about 30 minutes, then centrifuged at 5000 g (7500 rpm) at 4 °C for 5-10 min. We then carefully aspirated the supernatant and aliquot and stored them at -70 °C. This is the cell membrane/organelle protein extract. The nucleoprotein was then extracted. For adherent cells, we added 0.5 mL of ice-cold extraction buffer 3 and 5 μL of the protease inhibitor to each T25 culture flask (approximately 1 × 10^7^ cells), and placed the ice bath cells on a decolorization shaker for about 30 minutes at medium speed, then centrifuged at 6780 g (9000 rpm) at 4 °C for 10 min. We then carefully aspirated the supernatant and stored it at –70 °C. This is the nucleoprotein extraction solution. Finally, the cytoskeleton protein extraction was performed. For adherent cells, we added 1 mL of the ice-cold wash buffer and 10 μL of the protease inhibitor to each T25 culture flask (approximately 1 × 10^7^ cells) for washing. We discarded the supernatant, dissolve with 0.5 mL of extraction buffer 4, and repeatedly blew with a small caliber pipette tip until it was no longer very sticky, then stored it at -70 °C.

**Plasmid construction.** Plasmid construction was performed as reported previously (2). For the overexpression assay and the localization assay, the expression vector and fluorescence-labeled vector were constructed. In brief, the ACTN3/ARF1/PAT/ACSL3/LPCAT1 CDS region was amplified by the cDNA library of HepG2/C2C12 cells using KOD-Plus-Neo DNA polymerase (#KOD-401, TOYOBO, Shanghai, China). After gel extraction, the gene CDS fragment was cloned into the digested pcDNA3.1/pCMV-N-FLAG vector (digestion sites HindIII and BamHI) using a seamless cloning kit (#C112-01, ClonExpress II One Step Cloning Kit, Vazyme, Nanjing, China). For the localization assay, the gene CDS region was cloned into the digested pCMV-C-DsRed (#D2624, Beyotime Biotechnology, Nanjing, China) or pCMV-C-EGFP (#D2626, Beyotime Biotechnology, Nanjing, China).

**qPCR assay.** Real-time PCR was performed as reported previously^2^ using the QuantStudio 6 Flex Real-Time PCR System (ABI, Thermo Fisher, Shanghai, China) and Roche LightCycler® 480 (Roche, Switzerland), and the following PCR program: Denaturation at 95 °C for 10 min; amplification for 45 cycles at 95 °C for 15 s; annealing and extension at 60 °C for 1 min. A 2× SYBR Green qPCR Master Mix (#B21203, Bimake, Shanghai, China) was used for the RT-qPCR. Specific amplifications for certain PCR reactions were assessed using a melting curve. One negative control reaction, in which the cDNA template was replaced by water, was performed to avoid potential contamination. The sample from each well was repeated three times, and the comparative Ct (2^−ΔΔCt^) value method was used for relative quantification. Here, GAPDH (NM_002046.6) was used as the reference gene.

**Silver staining.** Silver staining was performed according to the manual (#P0017S, Beyotime, Nanjing, China). Briefly, the page glue was first fixed with ethanol/the acetic acid fixing solution at room temperature for 1 h. After that, it was washed with 30% ethanol and deionized water. After sensitization and water washing, silver staining was performed. After 1.5 minutes of washing with water, color developed, and the reaction was terminated after the expected band was observed.

**Immunoprecipitation and co-immunoprecipitation.** Immunoprecipitation and nuclear co-precipitation were performed according to the manufacturer’s instructions (Protein A/G beads, #B23201, bimake, Shanghai, China). An antigen sample was first prepared. We removed the culture medium and washed with 1 × PBS twice at a ratio of 150 μL per 1.0 × 10^5^ cells. We scraped the cells with a cell scraper and collected them into a 1.5 mL EP tube. Here, we used 20-30 μL per 1.0 × 10^5^ cells. We added the binding buffer and protease inhibitor at the same ratio, mixing and placing on ice for 10 min. We collected the supernatant by centrifugation (4 °C, 14,000 g, 10 min) and kept it on ice for later use. We vortexed the magnetic beads for 1 minute to fully suspend them, then took 25-50 μL of the magnetic bead suspension into a 1.5 mL EP tube. We added 200 μL of the binding buffer for washing and magnetic separation (placing the centrifuge tube on a magnetic stand and letting it stand for 2 minutes or waiting for the magnetic beads to adhere to the tube wall). We then aspirated and discarded the supernatant. We repeated the washing once, inserted the magnetic strip, then magnetically separated and aspirated the supernatant. We added 200 μL of the binding buffer to resuspend the beads for later use. We prepared the antibody working solution, diluted the antibody sample with the binding buffer, then prepared the antibody working solution with a final concentration of 5-50 μg/mL and kept it on ice for later use. We magnetically separated the pre-treated magnetic bead suspension and aspirated and discarded the supernatant. We then added 200 μL of the antibody working solution, resuspended it quickly, placed it in a mixing mixer at room temperature or gently inverted the EP tube manually, and performed magnetic separation after 15 min. We collected the supernatant and placed it on ice for subsequent testing. We removed the magnetic strip, added 200 μL of the binding buffer to the EP tube, and gently pipetted to disperse the magnetic bead-antibody complex evenly. We then inserted the magnetic strip and performed magnetic separation. We aspirated and discarded the supernatant, washing once. We added 200 μL of the antigen sample prepared in step 1 and gently pipetted to disperse the antigen and magnetic bead-antibody complex. We place it in an inversion mixer or gently inverted the EP tube by hand and reacted it at 4 °C overnight. We magnetically separated the magnetic bead-antibody-antigen complexes for antigen adsorption, as described above, collecting the supernatant and placing it on ice for subsequent detection. We removed the magnetic strip, added 200 μL of the washing buffer to the EP tube, and gently pipetted to disperse the magnetic bead-antibody-antigen complex uniformly. We inserted the magnetic strip and magnetically separated it, aspirating and discarding the supernatant. We repeated washing two times. We removed the magnetic strip, added 200 μL of the washing buffer, transferred the magnetic bead-antibody-antigen complex suspension to a new 1.5 mL EP tube, inserted the magnetic strip and performed magnetic separation, then aspirating the supernatant. Finally, the antigen was eluted, and the magnetic strip was drawn out. Here, 20-50 μL of the 1 × SDS-PAGE loading buffer was added to the mixture, and the mixture was heated at 95 °C for 5 minutes. We inserted the magnetic strip and performed magnetic separation to collect the supernatant for SDS-PAGE detection. All analyses were done with three biological replications (three wells of cells per replication).


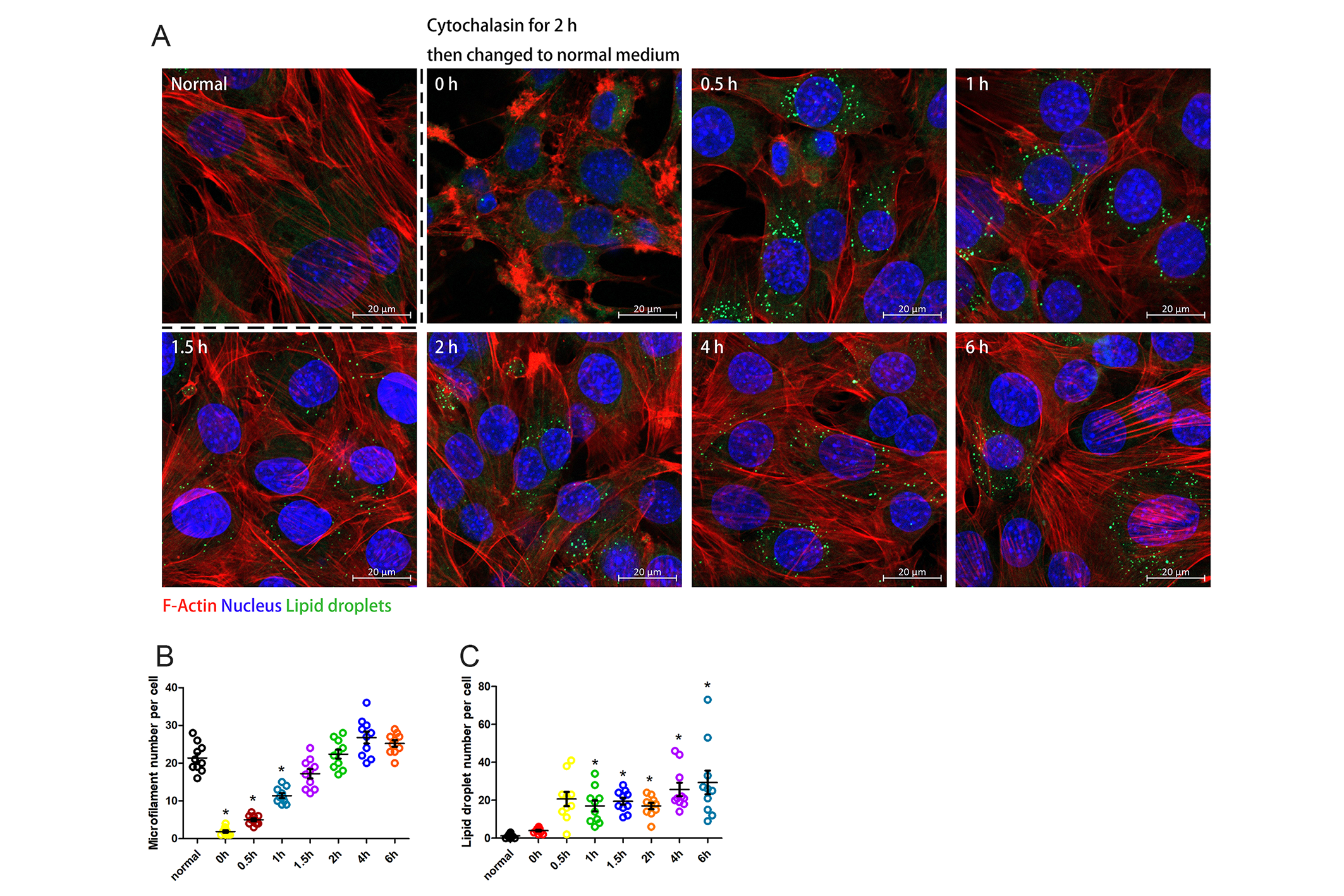


Fig. S1. LDs accelerate microfilament remodeling. A. C2C12 cells were treated with cytochalasin for 2 h, then the medium was replaced with a fresh medium. The microfilaments and LDs were marked and observed at 0, 0.5, 1, 1.5, 2, 4, and 6 h, respectively. B. The microfilament number per cell in A. C. The LD number per cell in A. *, p<0.05. Results are from three technical repeats (n=3) for a representative of three biological repeats (N=3).


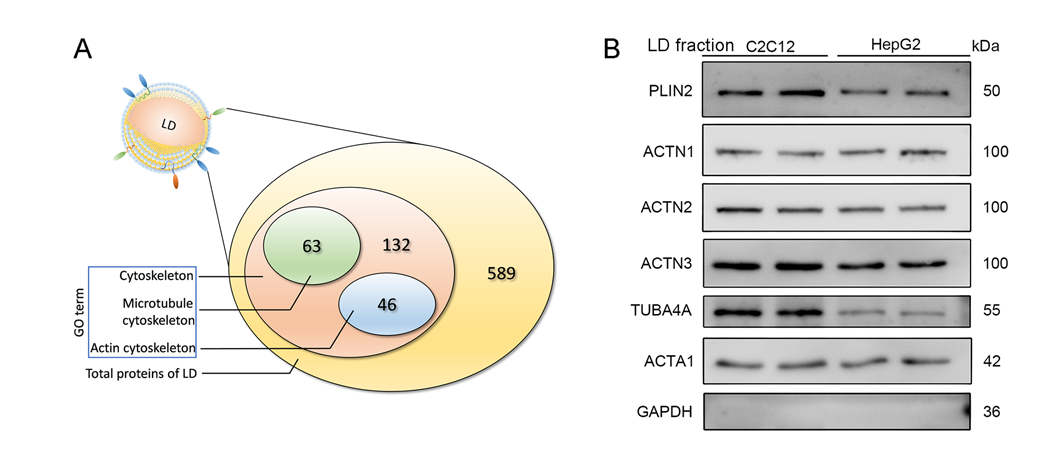


Fig. S2. LD proteomic data show many actinin proteins on LDs. Proteomic data showing many cytoskeleton-related proteins on lipid droplets (LDs). A. Protein mass spectrometry data indicating that LDs contain many cytoskeleton-related proteins. B. The cytoskeleton-related proteins on LDs were verified by Western blotting.


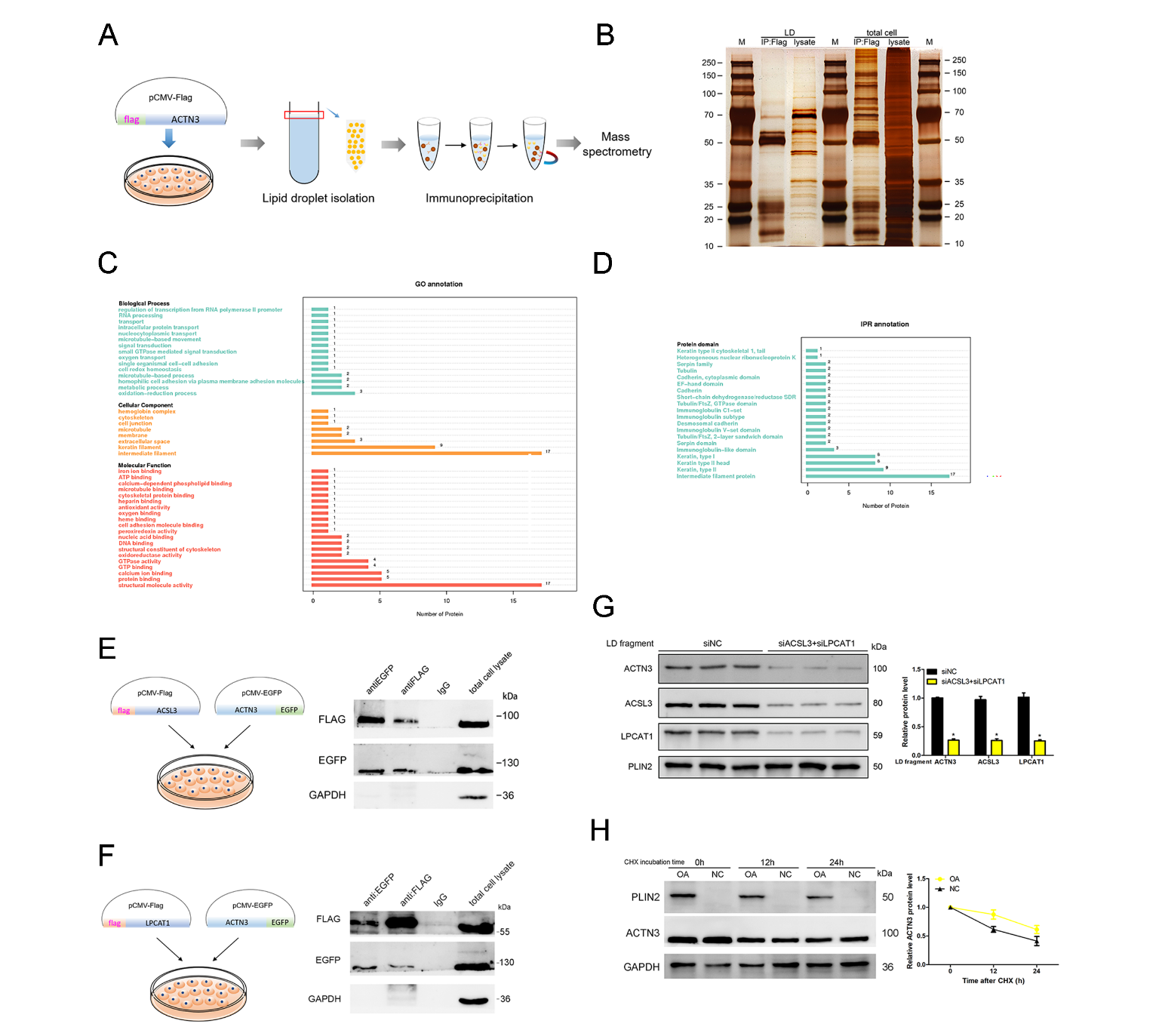


Fig. S3. LD proteins ACSL3 and LPCAT1 recruit actinin by binding SR protein domains. A. Protocol of the assay. Briefly, FLAG-ACTN3 was expressed in HepG2 cells, then the LDs were isolated, and the LD proteins were purified. The immunoprecipitation was performed with the protein, then the obtained proteins were analyzed by mass spectrometry. B. The proteins for mass spectrometry were detected by silver staining. C. GO cluster analysis of the mass spectrometry data. D. The protein domain analysis. E. Co-IP verification of ACTN3-ACSL3 interaction by the FLAG and EGFP tags. F. Co-IP verification of ACTN3-LPCAT1 interaction by the FLAG and EGFP tags. G. The expression levels of ACSL3 and LPCAT1 were knocked down by siRNAs, then the cellular LDs were isolated for the detection of ACTN3 levels. H. The detection of the half-life of ACTN3 in loaded (pre-incubated with OA medium) and unloaded cells (pre-incubated with BSA medium). *, p<0.05. Results are from three technical repeats (n=3) for a representative of three biological repeats (N=3).


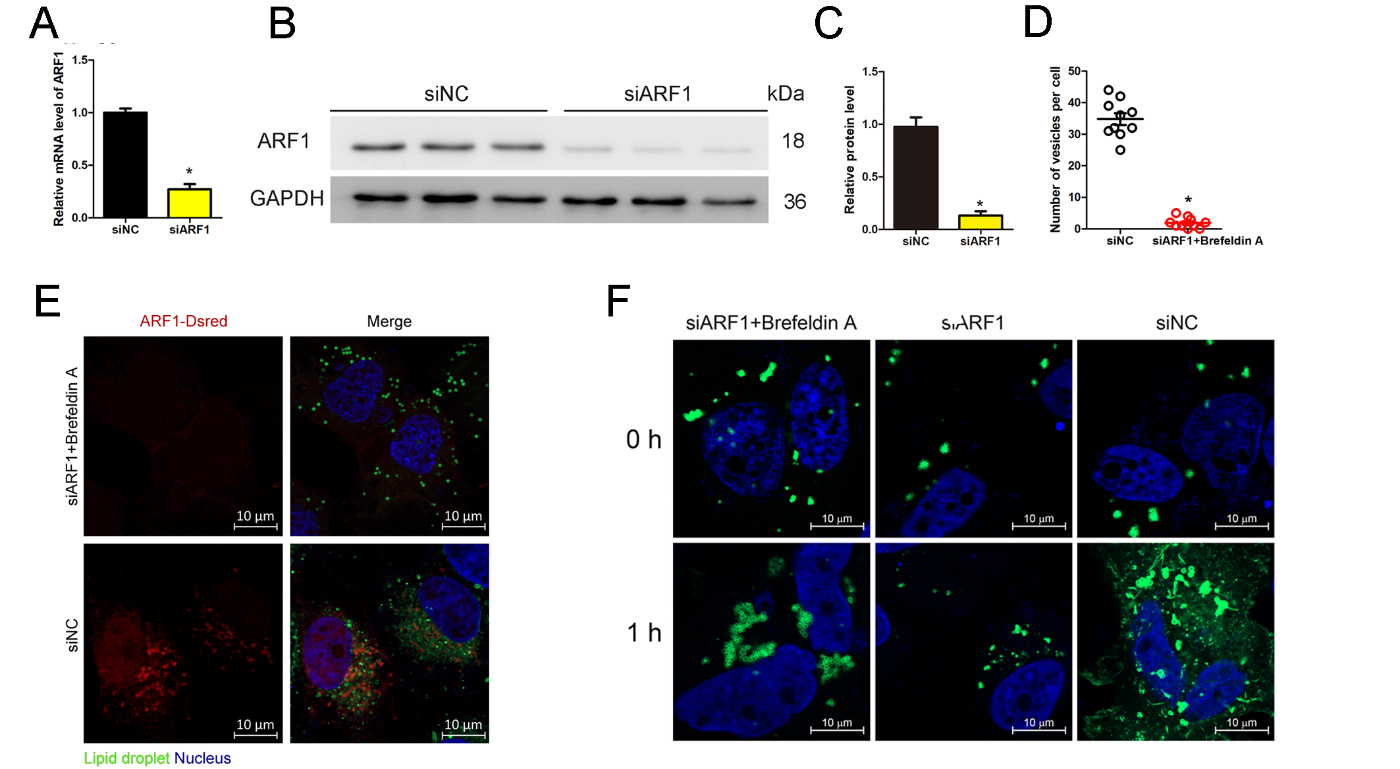


**Fig. S4.** ARF1-dependent vesicles mediate the transfer of actinin from LDs to microfilaments. A. qPCR assay showed ARF1 mRNA levels were decreased by siRNAs. B. Western blotting showed ARF1 protein levels were decreased by siRNAs. C. Gray value analysis of western blotting. D. The number of vesicles per cell in siARF1 + brefeldin A treatment cells and siNC cells. E. Fluorescence detection of ARF1-DsRed in siARF1 + brefeldin A treatment cells and siNC cells. F. The cells were treated with siARF1 + brefeldin A or siARF1 or siNC, then the cells were treated with cytochalasin for 2 h, then the medium was replaced with a fresh medium. The PAT-ACTN3-EGFP signals were detected at 0 and 1 h, respectively. The results of E and F derived form HepG2. *, p<0.05. Results are from three technical repeats (n=3) for a representative of three biological repeats (N=3).


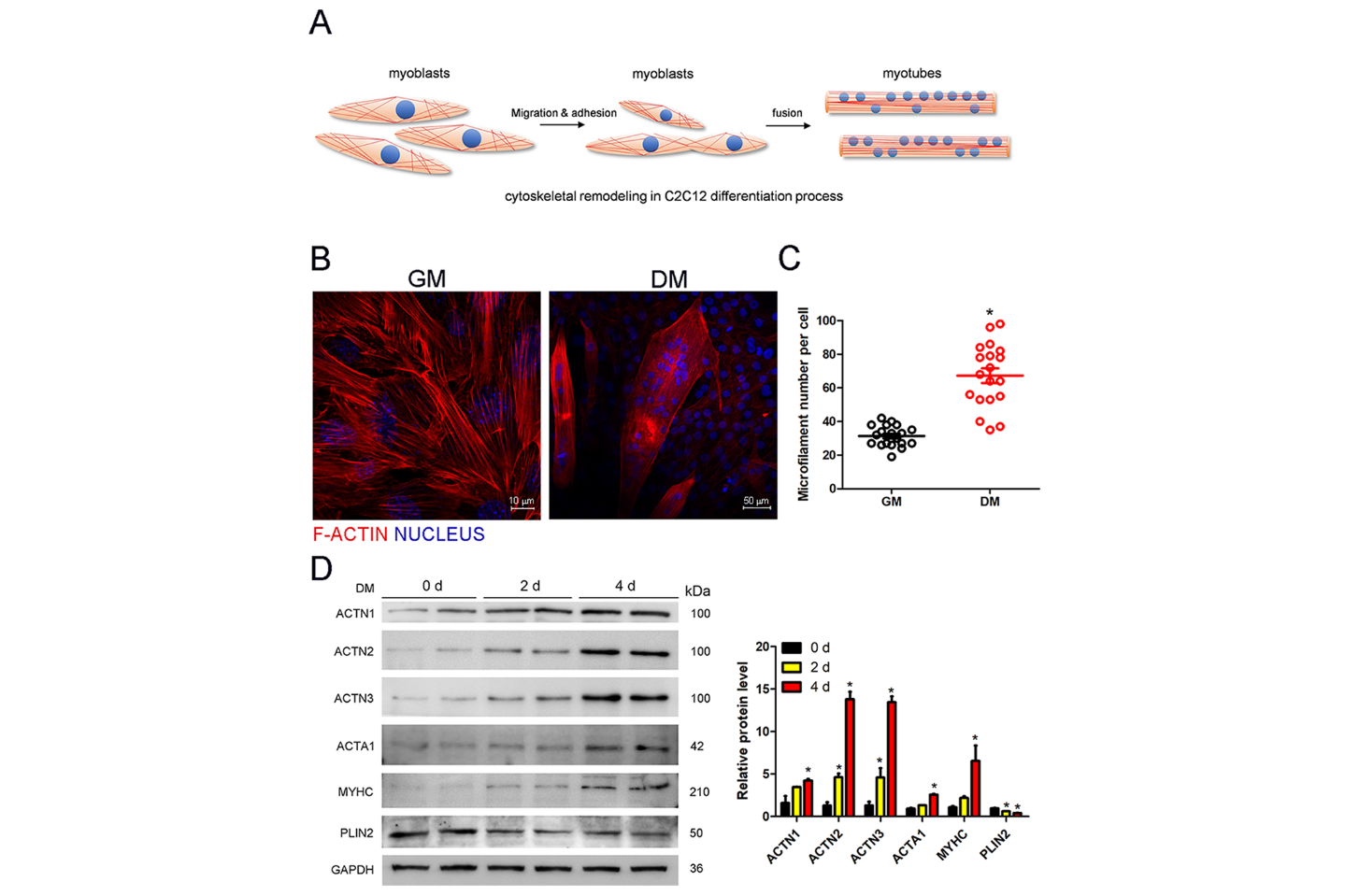


**Fig. S5.** Microfilament remodeling during C2C12 differentiation. A. The schematic diagram of the changes of microfilaments during C2C12 differentiation. B. Microfilaments in myoblasts and myotubes. C. The number of microfilaments per cell. D. The cytoskeleton-related protein levels during C1C12 differentiation.

Table S1. Primer used for SYBR Green I qRT-PCR validation.

| **Gene symbol** | **Primer Sequence 5′-3′** | **Tm**℃ |
| --- | --- | --- |
| ***mMYHC*** | Forward: CAAGTCATCGGTGTTTGTGG | 59 |
|  | Reverse: TGTCGTACTTGGGCGGGTTC |  |
| ***mACTN3*** | Forward: AACAGCAGCGGAAAACCTTCA | 59 |
|  | Reverse: GGCTTTATTGACATTGGCGATTT |  |
| ***EGFP*** | Forward: TTCAAGGACGACGGCAACTACAAG | 59 |
|  | Reverse: CCTTCAGCTCGATGCGGTTCAC |  |
| ***mMYOD*** | Forward: CCACTCCGGGACATAGACTTG | 59 |
|  | Reverse: AAAAGCGCAGGTCTGGTGAG |  |
| ***mMYOG*** | Forward: GAGACATCCCCCTATTTCTACCA | 59 |
|  | Reverse: GCTCAGTCCGCTCATAGCC |  |
| ***mMYOMAKER*** | Forward: TTCCTCCCGACAGTGAGCAT | 59 |
|  | Reverse: GCACAGCACAGACAAACCAG |  |
| ***mCAVEOLIN*** | Forward: ATGTCTGGGGGCAAATACGTG | 59 |
|  | Reverse: CGCGTCATACACTTGCTTCT |  |
| ***hGAPDH*** | Forward: CTGGGCTACACTGAGCACC | 59 |
|  | Reverse: AAGTGGTCGTTGAGGGCAATG |  |
| ***hARF1*** | Forward: ATGGGGAACATCTTCGCCAAC  Reverse: GTGGTCACGATCTCACCCAG | 59 |
| ***mARF1*** | Forward: TGGGCGAAATTGTGACCACC | 59 |
|  | Reverse: TCCACTACGAAGATCAAGCCT |  |
| ***mGAPDH*** | Forward: AGGTCGGTGTGAACGGATTTG | 59 |
|  | Reverse: TGTAGACCATGTAGTTGAGGTCA |  |
| ***hACTN3*** | Forward: GTACCGCAACGTCAACGTG | 59 |
|  | Reverse: CGTAGTCGATGAGGTCAGGG |  |

Movie S1 (separate file). The detection of cell migration capacity in the loaded and unloaded cells by a live cell workstation.

**SI References**

Sample References:

1. Lv Y*, et al.* (2016) Deep sequencing of transcriptome profiling of GSTM2 knock-down in swine testis cells. *Scientific Reports* 6:38254.

2. Tan Y*, et al.* (2019) Perilipin 5 Protects against Cellular Oxidative Stress by Enhancing Mitochondrial Function in HepG2 Cells. *Cells* 8(10).
